# Characterization of a novel bioflocculant from a marine bacterium and its application in dye wastewater treatment

**DOI:** 10.1186/s12896-017-0404-z

**Published:** 2017-11-17

**Authors:** Zhen Chen, Zhipeng Li, Peize Liu, Yu Liu, Yuanpeng Wang, Qingbiao Li, Ning He

**Affiliations:** 1 Department of Chemical and Biochemical Engineering, College of Chemistry and Chemical Engineering, Xiamen University, Xiamen, 361005 People’s Republic of China; 2 The Key Lab for Synthetic Biotechnology of Xiamen City, Xiamen University, Xiamen, 361005 People’s Republic of China; 3 College of Life Sciences, Xinyang Normal University, Xinyang, Henan 464000 China

**Keywords:** Bioflocculant, *Alteromonas* sp., Marine bacterium, Dye wastewater

## Abstract

**Background:**

The identification of microorganisms with excellent flocculant-producing capability and optimization of the fermentation process are necessary for the wide-scale application of bioflocculants. Thus, we evaluated the flocculant-producing ability of a novel strain identified by the screening of marine bacteria, and we report for the first time the properties of the bioflocculant produced by *Alteromonas* sp. in the treatment of dye wastewater.

**Results:**

A bioflocculant-producing bacterium was isolated from seawater and identified as *Alteromonas* sp. CGMCC 10612. The optimal carbon and nitrogen sources for the strain were 30 g/L glucose and 1.5 g/L wheat flour. In a 2-L fermenter, the flocculating activity and bioflocculant yield reached maximum values of 2575.4 U/mL and 11.18 g/L, respectively. The bioflocculant was separated and showed good heat and pH stability. The purified bioflocculant was a proteoglycan consisting of 69.61% carbohydrate and 21.56% protein (wt/wt). Infrared spectrometry further indicated the presence of hydroxyl, carboxyl and amino groups preferred for flocculation. The bioflocculant was a nanoparticle polymer with an average mass of 394,000 Da. The purified bioflocculant was able to remove Congo Red, Direct Black and Methylene Blue at efficiencies of 98.5%, 97.9% and 72.3% respectively.

**Conclusions:**

The results of this study indicated that the marine strain *Alteromonas* sp. is a good candidate for the production of a novel bioflocculant and suggested its potential industrial utility for biotechnological processes.

**Electronic supplementary material:**

The online version of this article (10.1186/s12896-017-0404-z) contains supplementary material, which is available to authorized users.

## Background

Flocculation is considered an easy, low-cost and eco-friendly separation process [1] and is carried out in a variety of industrial processes, including wastewater refinement, processes in the food-related and fermentation industries, and drinking water purification [2, 3]. Bioflocculants are natural macromolecular polymers produced by microorganisms that are capable of flocculating various suspended solids, such as cells and colloidal solids, with the character of harmless and biodegradable [4]. Therefore, bioflocculants have been extensively employed in removing pollutants (such as dye particles [5], heavy metal ions [6] and arsenite [7]) from wastewater, for sludge thickening and dewatering [8, 9] and for the harvesting of microbial biomass [10]. Bioflocculant-producing microorganisms have been isolated from a wide variety of ecosystems such as wastewater, rivers, soil and activated sludge [11]. In general, biological flocculation is a dynamic process, which often occurs in the aerobic treatment of activated sludge. Thus, activated sludge is considered one of the best and most favoured sources of bioflocculant-producing strains. A variety of microorganisms including *Chryseobacterium daeguense* [12], *Rhodococcus erythropolis* [13] and *Solibacillus silvestris* [14] have been isolated from activated sludge. Recently, some microorganisms that produce bioflocculants have also been isolated from unusual environments such as sputum [15] and human saliva [16].

Although many bioflocculants have been idntified, their large-scale production is still limited by low yield, high production cost, and week activity [3]. Thus, the search for microorganisms with better bioflocculant-producing capacities and the optimization of medium constituents and fermentation conditions are still effective strategies to improve bioflocculant yields and flocculating activity. Recent efforts to reduce the production cost of bioflocculants have been effective but not sufficient. The utilization of inexpensive substrates for bioflocculant production has been investigated. *Pseudomonas veronii* can produce a bioflocculant from the hydrolyzate of peanut hull, which can effectively save the production cost of bioflocculant [17]. Other agricultural wastes, such as rice stover and corn stover, have also been applied as inexpensive carbon sources to produce bioflocculants [18, 19]. Various wastewaters, including potato starch wastewater and chromotropic acid wastewater, have been used as cheap carbon sources to reduce the production cost [9, 20]. Other reports focus on the optimization of the culture media and conditions for bioflocculant production [7, 21]. Marine habitats, that support a rich biodiversity of marine bacteria, remain underexplored for industrial utilization and yet possess enormous potentials for screening novel bioflocculant-producing microorganisms. Due to their species diversity, marine microorganisms can produce a wide variety of metabolites with various structures [22]. In recent years, research on the abilities of marine microbes to secrete bioflocculant is receiving increasing attention [23, 24].

In this study, a marine bacterium, *Alteromonas* sp. CGMCC 10612, with excellent bioflocculant-producing capability was isolated, and a novel proteoglycan bioflocculant was identified. Subsequently, the actual applications of this bioflocculant in the treatment of various dye wastewaters were investigated under a variety of conditions. According to our literature search, no previous report has documented the use of *Alteromonas* sp. in the production of bioflocculant.

## Methods

### Isolation and identification of Bioflocculant-producing strains

Bioflocculant-producing strains were isolated from the surface seawater collected from the SEATS station in the South China Sea (18°N, 116°E). The enrichment culture was set up with 2% (*v*/v) seawater in a medium containing (g/L) tryptone 10.0, yeast extract 5.0, nutrient broth 0.5, sea salt 34.0, sodium citrate 0.5, sodium acetate 1.0 and NH_4_NO_3_ 0.2 (pH 7.5). The enrichment test was performed under aerobic conditions on a rotary shaker at 30 °C and 150 rpm. After a 24-h incubation, 1 mL of culture broth was inoculated to the same medium three times to enrich the microbial culture.

The enrichment culture was diluted and spread onto agar plates containing the following sterilized medium (g/L): glucose 10.0, urea 1.0, yeast extract 1.0, sea salt 34.0, KH_2_PO_4_ 0.1 and K_2_HPO_4_ 0.1 (pH 7.5). After cultivation for 2 days at 30 °C, single colonies with mucoid and ropy morphology were picked and inoculated into liquid for 48 h at 30 °C and 150 rpm. The strains with the ability to produce bioflocculant were selected and spread onto agar plates for 48 h at 30 °C, followed with 5 cycles of agar plate coating to ensure the purities of the strains with the highest flocculating activity.

The bioflocculant-producing strains were identified on the basis of the 16S rRNA gene according to the method in a previous study [17]. The genomic DNA from *Alteromonas* sp. CGMCC 10612 was extracted using an E.Z.N.A. Bacterial DNA Kit (OMEGA, Norcross, GA). The 16S rRNA gene was sequenced by Sangon (China) and analysed by blast in the National Center for Biotechnology Information (NCBI) Database.

### Determination of flocculating activity

Flocculating activity was determined using kaolin-clay suspensions as an indicator as described previously [25]. Each sample was analysed in triplicate. A control was performed with uninoculated culture medium substituted for the sample.

### Effects of initial pH, temperature, sources of carbon and nitrogen and metal ions on the Bioflocculant production

*Alteromonas* sp. CGMCC 10612 was selected for further experimental investigation to optimize the process parameters. Except as otherwise noted, all liquid cultures were grown in triplicate in 250-mL flasks containing 50 mL of medium on a rotary shaker at 30 °C and 150 rpm. To obtain the optimum fermentation temperature, the bacteria were cultured at 20, 25, 30, 37 and 42 °C, and then the flocculating activity and OD_600_ of the 48-h broth were determined. The effects of pH variation in the range of 4.0–10.0 on the cell growth and bioflocculant production were also analysed. Bioflocculant production was also monitored using various carbon sources such as glucose, sucrose, starch, fructose, glycerol, lactose and sodium citrate at 10 g/L. The impact of various organic and inorganic nitrogen sources such as yeast extract, tryptone, beef extract, soy flour, wheat flour, urea, NaNO_3_ and NH_4_Cl was also explored when the medium contained glucose as the carbon source and the initial pH was 7.5. Furthermore, the effects of different proportions of phosphate as well as different concentrations of sea salt, glucose and wheat flour on the production of the bioflocculant from *Alteromonas* sp. CGMCC 10612 were investigated.

### Culture process in a 2-L fermenter

Fermentation was carried out in a 2-L fermenter (ez-Control, made in Holland) containing 1.5 L of fermentation medium with an inoculum of 50 mL. The culture was carried out at 37 °C, and DO was automatically controlled to remain no lower than 30%. Samples were taken every 4 h and then subjected to further analysis.

### Purification of the Bioflocculant

The fermentation broth was centrifuged at 12,000 rpm for 10 min to remove bacteria. The supernatant was then mixed with 3 volumes of chilled ethanol and left to stand at 4 °C overnight. The resultant precipitate was collected by centrifugation at 8000 rpm for 15 min, and the crude bioflocculant was obtained. The crude bioflocculant was redissolved in distilled water, followed by dialysis using a membrane with a 7000–14,000 MWCO at 4 °C for 12 h. Three volumes of cold ethanol were then added. After 2 h, the resulting precipitate was collected by centrifugation at 8000 rpm for 15 min and finally lyophilized to collect the purified bioflocculant.

### Characterization of purified bioflocculant

#### Compositional analysis of purified bioflocculant

The total protein content and sugar content of the purified bioflocculant were determined by the Lowry method using bovine serum albumin as the standard solution and the phenol-sulfuric acid method using glucose as the standard solution, respectively. The purified bioflocculant was hydrolyzed with trifluoroacetic acid at 121 °C for 2 h to obtain the component sugars. The resultant amino sugars, neutral sugars and uronic acid content were determined using the Elson-Morgan method, the anthrone reaction method and the carbazole-sulfuric acid method [26].

#### Elementary analysis

Carbon, hydrogen and nitrogen were analysed using an Elemental Analyzer (Vario EL III). For this purpose, 10 mg of freeze-dried bioflocculant was placed in tin cups, and the mode of operation was selected as CHN.

#### FTIR spectroscopy of purified bioflocculant

The functional groups of purified bioflocculant were determined using a Fourier transform infra-red (FT-IR) spectrophotometer (Thermo Electron Corporation, USA) over a wavenumber range of 4000–500 cm^−1^.

#### Molecular weight determination of purified bioflocculant

The molecular weight of the bioflocculant was determined by high-performance gel permeation chromatography (HPGPC) coupled to refractive index (RI) detection as described previously [25].

#### Scanning electron microscopy (SEM) imaging

The purified bioflocculant was re-dissolved in the purified water as the samples. The samples were placed on a silicon wafer and gold coated in a gold-coating chamber using an Eiko IB.3 ION coater. Scanning electron microscopy (SEM) images of the bioflocculant were obtained using an FEI XL30 (FEI; Netherlands).

#### Stability analysis of purified bioflocculant

To examine the thermal stability of the bioflocculant, the purified bioflocculant was incubated at 100 °C for different times (15, 30, 45 and 60 min). To investigate the effect of pH on flocculating activity, the pH of the kaolin-clay suspensions were adjusted to the pH range of 3–11 using HCl or NaOH.

### Coagulation–flocculation experiments

First, 1.0 mL of bioflocculant was added to 99 mL of dye solution (100 mg/L). The coagulation procedure was as follows: rapid mixing (200 rpm) for 1.0 min followed by slow mixing (100 rpm) for 10 min and then a transfer into a 100-mL measuring cylinder and sedimentation for 60 min. After flocculation, the supernatants were collected at 1 cm below the wastewater surface and filtered through a slow Whatman filtration membrane, then analysed by a UV-visible spectrophotometer at the maximum adsorption wavelength. The colour removal efficiency was calculated as follows:

Dye removal efficiency (%) = (C_0_-Ce)/C_0_ × 100%.

where C_0_ and Ce were the initial and final concentrations of the dye solution, respectively.

## Results and discussion

### Isolation of Bioflocculant-producing bacterium

Approximately 285 bacterial isolates were obtained from seawater samples, and 32 isolates were selected to be screened for bioflocculant production. After three subcultures, only 4 strains were able to actively flocculate kaolin suspension, as measured. Among them, the bacterium named H-6 with the highest flocculating activity (259.21 U/mL) was selected as the bioflocculant-producing bacterium for further study and is currently preserved at the China General Microbiological Culture Collection Centre (registration number is CGMCC 10612). The flocculating efficiency of strain H-6 against kaolin suspension before medium optimization could be up to 96%, which is much higher than that of recently reported strains such as *Achromobacter xylosoxidans* strain TERIL1 (83.3%) [27] and *Arthrobacter humicola* strain R1 (85%) [28].

### Identification and characterization of Bioflocculant-producing bacterium

The colonies of strain CGMCC 10612 were round in shape with a neat edge and a central uplift. The surface was smooth and translucent with a greyish-yellow colour. Cells of strain CGMCC 10612 were gram-negative, non-spore forming, short rod shaped and small in size. The physiological and biochemical characteristics of strain CGMCC 10612 are summarized in Table 1 and were basically consistent with *Alteromonas macleodii* [29]. Compared to the 16S rDNA sequences deposited in the NCBI GenBank database, the 16S rDNA Sequence of strain CGMCC 10612 is most similar to that of *Alteromonas* sp., sharing 99% similarity. A phylogenetic tree was constructed according to the neighbour-joining algorithm (see Additional file 1). Therefore, strain CGMCC 10612 was identified as *Alteromonas* sp. by both its morphological/physiological and its phylogenetic characteristics. The *Alteromonas* genus is widely distributed in marine environments. Related research has suggested that *Alteromonas* strains can disproportionately alter the fate of carbon in the mesotrophic ocean and act functionally in ecosystem [30]. There have been many reports that *Alteromonas* strains can produce extracellular polysaccharides [31–33]. However, *Alteromonas* sp. has not been reported as a bioflocculant-producing strain in previous studies.

**Table 1:** Biophysiological and biochemical properties of strain H-6

Test	Strain H-6	*Alteromonas macleodii*
Starch hydrolysis	+	+
Catalase	+	+
H_2_S production	+	+
Gelatin hydrolysis	+	+
Indole test	–	0
Methyl-red test	–	+
Voges-Proskauer test	–	–
Citrate utilization	–	0
Arginine utilization	+	0
Phenylalanine utilization	–	0
Glucose utilization	+	+
Lactose utilization	+	+
Sucrose utilization	+	+
Gram reaction	G^−^	G^−^

(+) Positive reaction, (−) Negative reaction, (0) No test

### Optimization of culture conditions for Bioflocculant production

#### Effect of temperature and initial medium pH on Bioflocculant production

Temperature and pH play important roles in the bacterial growth rate and enzymatic activity and thus impact the production of bioflocculants [34]. The effects of temperature and variation of the initial pH in the range of 4–10 on bioflocculant production by strain CGMCC 10612 were investigated (see Additional file 2). The optimal temperature for the bioflocculant production of strain CGMCC 10612 was found to be 25 °C, at which point OD_600_ reached its highest value. The flocculation activity decreased sharply to approximately 200 U/mL at 30 °C. The lower flocculation activity of strain CGMCC 10612 at high temperature could be attributed to decreased enzyme activity and biomass. In the initial pH range of 7–8, considerable flocculating activity was observed, which indicated that production of this bioflocculant is appropriate to neutral and moderately alkaline conditions. The optimal initial pH was 7.5, at which the flocculating activity reached 600 U/mL.

#### Effect of carbon and nitrogen sources on Bioflocculant production

The importance of carbon and nitrogen sources for bioflocculant production has been emphasized [35, 36]. Carbon source plays a significant role in the growth of cells and the synthesis of varied metabolites during cultivation. Among the various carbon sources utilized, glucose exhibited the most prominent effect on the flocculating activity (1515 U/mL) followed by fructose (1120 U/mL). Starch and glycerol were conducive to the biomass accumulation of *Alteromonas* sp. but did not favour the production of bioflocculant. The carbon source requirements differ for different bacteria; for instance, glucose was preferred by *Proteus mirabilis* TJ-1 [37], while lactose was preferred by *S. ficaria* [38].

**Fig. 1:**
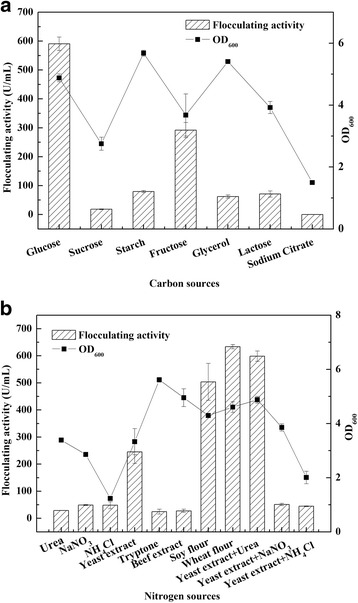
Effects of different carbon sources (**a**) and nitrogen sources (**b**) on bioflocculant production

The effect of different nitrogen sources on the bioflocculant production of strain CGMCC 10612 was investigated by employing glucose as the carbon source and is illustrated in Fig. 1b. It was observed that the flocculating activity obtained with inorganic nitrogen sources was comparatively low. The addition of organic nitrogen sources, such as soy flour, wheat flour and yeast extract, is essential for optimum bioflocculant production. These results were consistent with the conclusion reported by Sekelwa et al. that organic nitrogen sources were more conducive to produce bioflocculant than inorganic nitrogen sources [39]. Strain CGMCC 10612 could grow well and produce considerable bioflocculant with high flocculating activities when yeast extract mixed with urea, soy flour and wheat flour was used as a nitrogen source. Considering the flocculating activity and economic factors, wheat flour was selected as the optimum nitrogen source.

#### Effect of different proportions of phosphate on Bioflocculant production

**Fig. 2:**
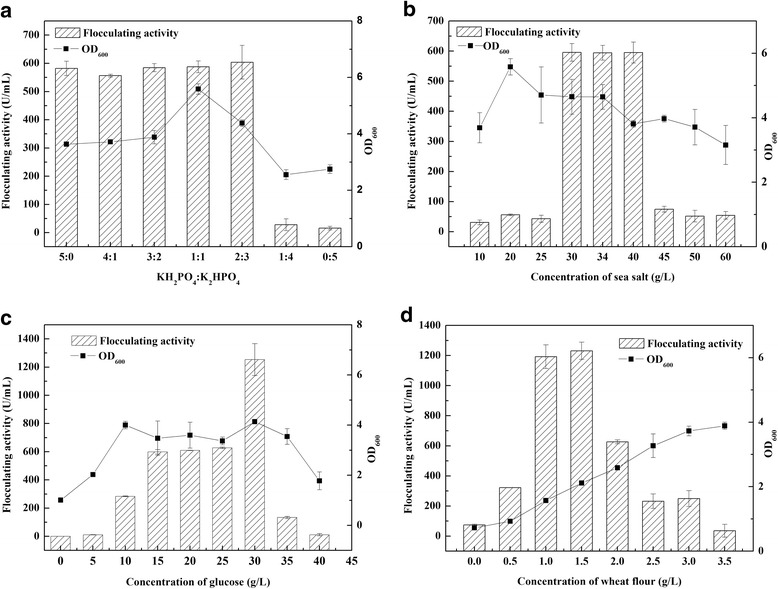
Effects of different concentrations of phosphate (**a**), sea salt (**b**), glucose (**c**) and wheat flour (**d**) on bioflocculant production

Phosphate can form a good buffer system, which plays an important role in the regulation of pH during fermentation. The properties of bioflocculant production by strain CGMCC 10612 were investigated. As shown in Fig. 2a, considerable bioflocculant could be produced by strain CGMCC 10612 when the proportion of phosphate ranged from 5:0 to 2:3. The biomass reached its highest value when the proportion of phosphate was 1:1. Considering the flocculating activity and microbial growth, a 1:1 proportion of phosphate was selected for further studies.

#### Effect of different concentrations of sea salt on Bioflocculant production

Sea salt not only provides the necessary salt-rich environment for marine bacteria to maintain osmotic pressure but also supplies the trace elements to promote microbial growth and bioflocculant production. Strain CGMCC 10612 was able to grow well in all salt concentration ranges tested, indicating that the strain possessed the property of adaptation to salinity variation (Fig. 2b). The highest flocculating activity was observed at the sea salt concentration of 30–40 g/L. Higher concentrations resulted in significant decreases in flocculating activity, which may be because microbial activity was inhibited by the hypersaline conditions.

#### Effect of different concentrations of glucose on Bioflocculant production

As a carbon source, glucose is an important factor for bacterial growth and the accumulation of secondary metabolites. As shown in Fig. 2c, the biomass and bioflocculant activity of strain CGMCC 10612 varied greatly at different concentrations of glucose. With increasing glucose concentration, the biomass increased to its highest value when the glucose concentration was 10 g/L, which was not sufficient to enhance the bioflocculant production. When the glucose concentration reached 30 g/L, the highest flocculating activity was obtained. Higher concentrations resulted in a significant decrease in flocculating activity, which may be due to the decline in biomass or to the accumulation of inactive by-products.

#### Effect of different concentrations of wheat flour on Bioflocculant production

The carbon/nitrogen source ratio can significantly influence the yield of bioflocculant because the C/N ratio greatly affects microbial metabolism [34]. Therefore, the effect of the concentration of wheat flour on flocculating activity was determined when the carbon source was 30 g/L glucose (Fig. 2d). The biomass increased gradually with increasing wheat flour concentration. The maximum bioflocculant production was achieved when the wheat flour concentration was 1.5 g/L. However, a further increase in wheat flour concentration caused a decline in bioflocculant production, indicating that the decline in the C/N ratio was not conducive to bioflocculant production.

### Scale-up of Bioflocculant production in a 2-L Fermenter

**Fig. 3:**
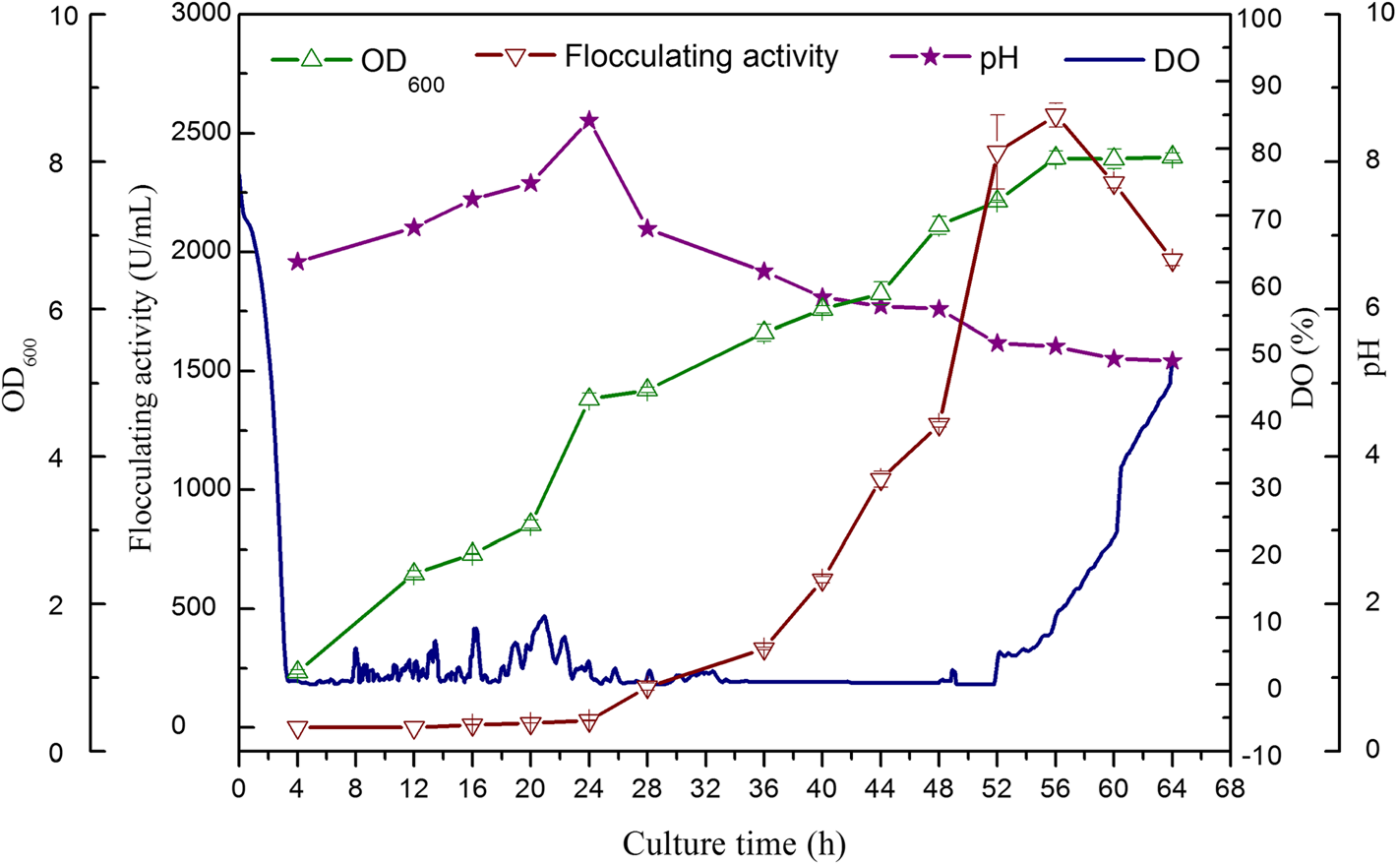
Batch production of the bioflocculant in a 2-L fermenter by *Alteromonas* sp. CGMCC 10612

After optimization of the fermentation conditions and medium, the ability of *Alteromonas* sp. CGMCC 10612 to produce bioflocculant during large-scale fermentation was investigated in a 2-L fermenter. A typical fermentation profile in terms of DO, pH, OD_600_ and flocculating activity is shown in Fig. 3. During the fermentation process, the agitation speed was kept at 150 rpm and the temperature at 25 °C, while the pH of the fermentation was not controlled but was monitored. The initial pH was approximately 6.8 to 7.0, and the pH to 5.3 at the end of the fermentation. The decrease in pH is thought to be attributable to the oxidation of glucose to gluconic acid. After inoculation, the dissolved oxygen gradually decreased, and after 4 h, OD_600_ appeared to undergo a sharp increase corresponding to rapid bacteria growth. It was observed that the flocculating activity increased progressively with increasing optical density of the culture, which signalled that the production of bioflocculant was positively associated with cell growth. The flocculating activity reached 2575.4 U/mL after 56 h of fermentation, which was 93.3% higher than the activity obtained during shake flask cultivation. The yield finally reached 11.18 g/L, which was 83.9% greater than the yield seen during shake flask cultivation.

In the shake flask, the bioflocculant production of most of the bioflocculant-produced strains, such as *Aspergillus flavus* [40], *Bacillus clausii* [41] and *Serratia ficaria* [38], was lower than 3 g/L. In this study, it was reported to be 6.08 g/L, which was much higher than the yields described in previous studies. Although bioflocculant production has been investigated in prior studies, the yields have been relatively low. By scaling up fermentation from a flask to a 10-L fermenter, HBF-3 bioflocculant production was increased to 5.58 g/L [42]. Patil S V et al. reported that the maximum EPS bioflocculant production was 6.10 g/L in a 2.5-L fermenter using optimized medium [43]. This result demonstrates that *Alteromonas* sp. CGMCC 10612 has great potential in the industrial production of bioflocculant. The bioflocculant production of strain CGMCC 10612 during scale-up may be further improved by optimizing the feeding strategy.

### Characterization of Bioflocculant

#### Composition analysis

Elemental analysis showed that the bioflocculant from *Alteromonas* sp. CGMCC 10612 had a C content of 20.49%, a H content of 4.48% and a N content of 5.54%. The Folin-Lowry results revealed that the purified bioflocculant consisted of 21.56% proteins. The phenol-sulfuric acid analysis to determine the total sugar showed that the bioflocculant consisted of 69.61% sugars, indicating that polysaccharides were the major component of the bioflocculant. Further analysis indicated that the mass proportion of neutral sugar, uronic acid and amino sugar was 2:1:1. Sufficient content of uronic acid in a bioflocculant molecule can provide carboxyl groups to the molecular chain, which are preferred for the adsorption of particles and for flocculation [44]. It has been proven that flocculation capacity was positively associated with uronic acid content [45].

#### Spectroscopic characterization

**Fig. 4:**
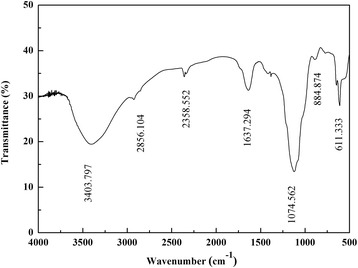
Infrared spectra of the purified bioflocculant

The FTIR spectrum was determined and showed the presence of hydroxyl, amide and carboxyl groups in the bioflocculant (Fig. 4). The spectrum showed an intense and broad absorption peak at 3403 cm^−1^, which implied the presence of a hydroxyl or amide group. A weak C-H stretching band was observed at 2856 cm^−1^ and is known to be typical of carbohydrates. A weak peak observed at 2358 cm^−1^ could be assigned to CO_2_ adsorption or to the amine group. An asymmetrical stretching peak at 1637 cm^−1^ could be attributed to the C = O stretching vibration in -NHCOCH_3_. A strong absorption band at 1074 cm^−1^ indicated the asymmetrical stretching vibration of a C-O-C ester linkage. A strong band at 884.87 cm^−1^ could be associated with the β-glycosidic linkage between the sugar monomers. In addition, a weak peak at 611 cm^−1^ could be due to the stretching of C-Br alkyl halides. The infrared spectrum showed characteristic peaks for carbohydrates and amides, which serves as further confirmation that the bioflocculant produced by strain CGMCC 10612 most likely belongs to the glycoprotein group.

#### Molecular weight and SEM analysis

**Fig. 5:**
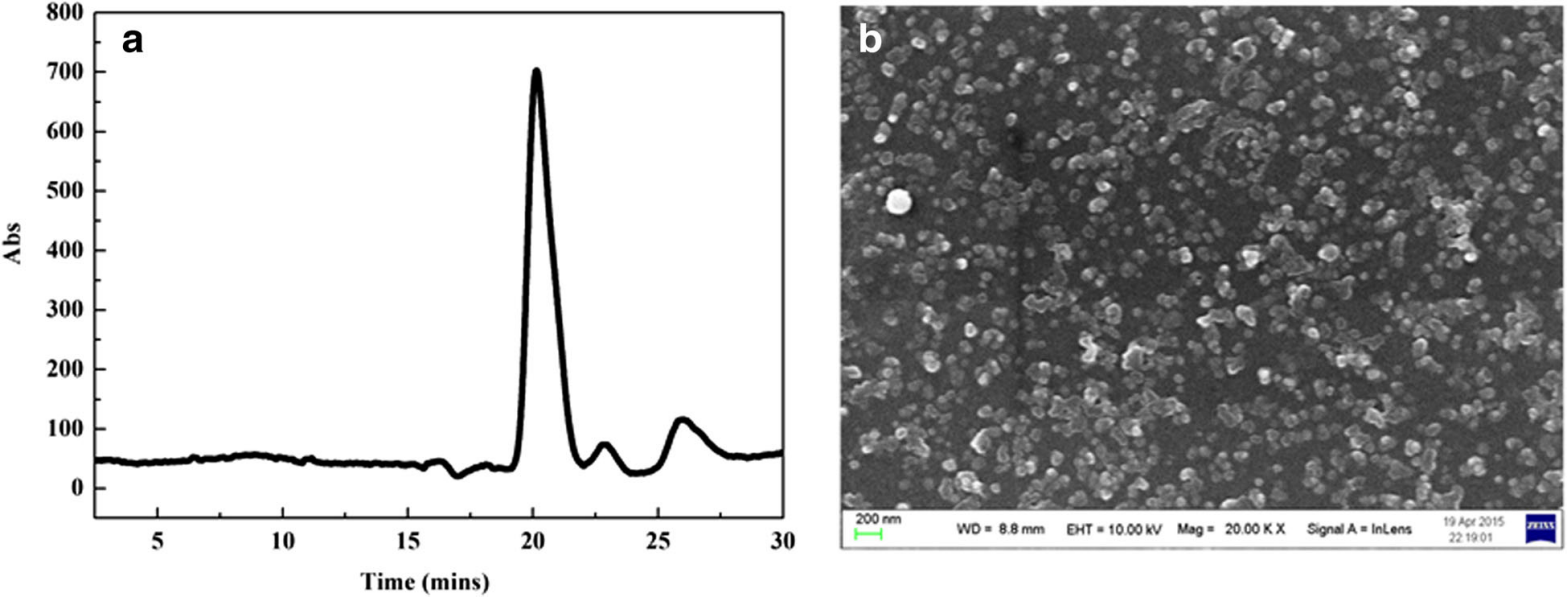
Gel permeation chromatogram (**a**) and SEM (**b**) of the purified bioflocculant

The HPGPC spectrum of the purified bioflocculant showed a symmetrical and sharp peak in the time of 20.556 min (Fig. 5a). The molecular mass-retention time equation accorded with the calibration curve was as follows: log (molecular weight) = −0.1368 T +8.3496. The average weight of the bioflocculant was calculated to be 3.94 × 10^5^ Da, which is much higher than the weight of other bioflocculants reported previously [36, 40, 46]. Bioflocculants with high molecular weight present stronger bridging, more adsorption points, and higher flocculating activities than those with low molecular weight [4].

SEM observation was carried out to determine the surface morphology of the purified bioflocculant (Fig. 5b). Micrograph images of the purified bioflocculant revealed nano-structured granules with an average size of 200 nm. The nanoparticle polymer was coarse-grained and varied in size. This property of the bioflocculant contributed not only to the flocculation of the kaolin-clay particles but also to sustained drug delivery, cancer chemotherapy and bioimaging [47].

#### Stability analysis of Bioflocculant

Investigation of the stability of the bioflocculant showed that the bioflocculant was relatively stable after a long period of heat treatment and at a wide range of pH values (Table 2). After heat treatment at 100 °C for 15 min, the bioflocculant exhibited good flocculating capability without loss of activity, which could satisfy the requirements for practical use in industry. Meanwhile, over 82% of the flocculating activity was maintained after heat treatment at 100 °C for 30 min. The bioflocculant was much more thermally stable than the recently reported polysaccharide bioflocculant, which decreased in activity by 65.2% after being heated at 80 °C for 30 min [48]. After heating at 100 °C for 60 min, the bioflocculant activity still reached 850 U/mL with a 34.6% decrease, indicating that the protein is one of the functional components. The optimum pH for different bioflocculants may vary due to their different compositions. The electric states of the bioflocculant vary for different pH, which in turn affect the flocculation ability of the bioflocculant for the kaolin particles. The highest flocculating activity of 1301.8 U/mL was attained at pH 9.0. The high flocculating activity achieved in a wide pH range suggests that this bioflocculant could be applied to treat various wastewaters in various industries without adjusting the pH, thus rendering the bioflocculant cost-effective. Similar findings were reported for the bioflocculant produced by *Bacillus pumilus*, which presented good flocculation ability under both acidic and alkaline conditions [49].

**Table 2:** Effects of heating time and pH on the flocculating activity of the purified bioflocculant (*n* = 3, mean ± SD)

Heating time (min)	FA ± SD (U/mL)	pH	FA ± SD (U/mL)
0	1313.1 ± 5.8	3	938.9 ± 27.8
15	1289.2 ± 16.0	5	1119.4 ± 25.6
30	1080.2 ± 35.5	7	1251.4 ± 36.5
45	926.1 ± 16.6	9	1301.8 ± 14.7
60	858.7 ± 19.4	11	1014.3 ± 35.6
		13	695.9 ± 69.4

### Decolorization by the Bioflocculant

#### Effect of solution pH on dye removal

**Fig. 6:**
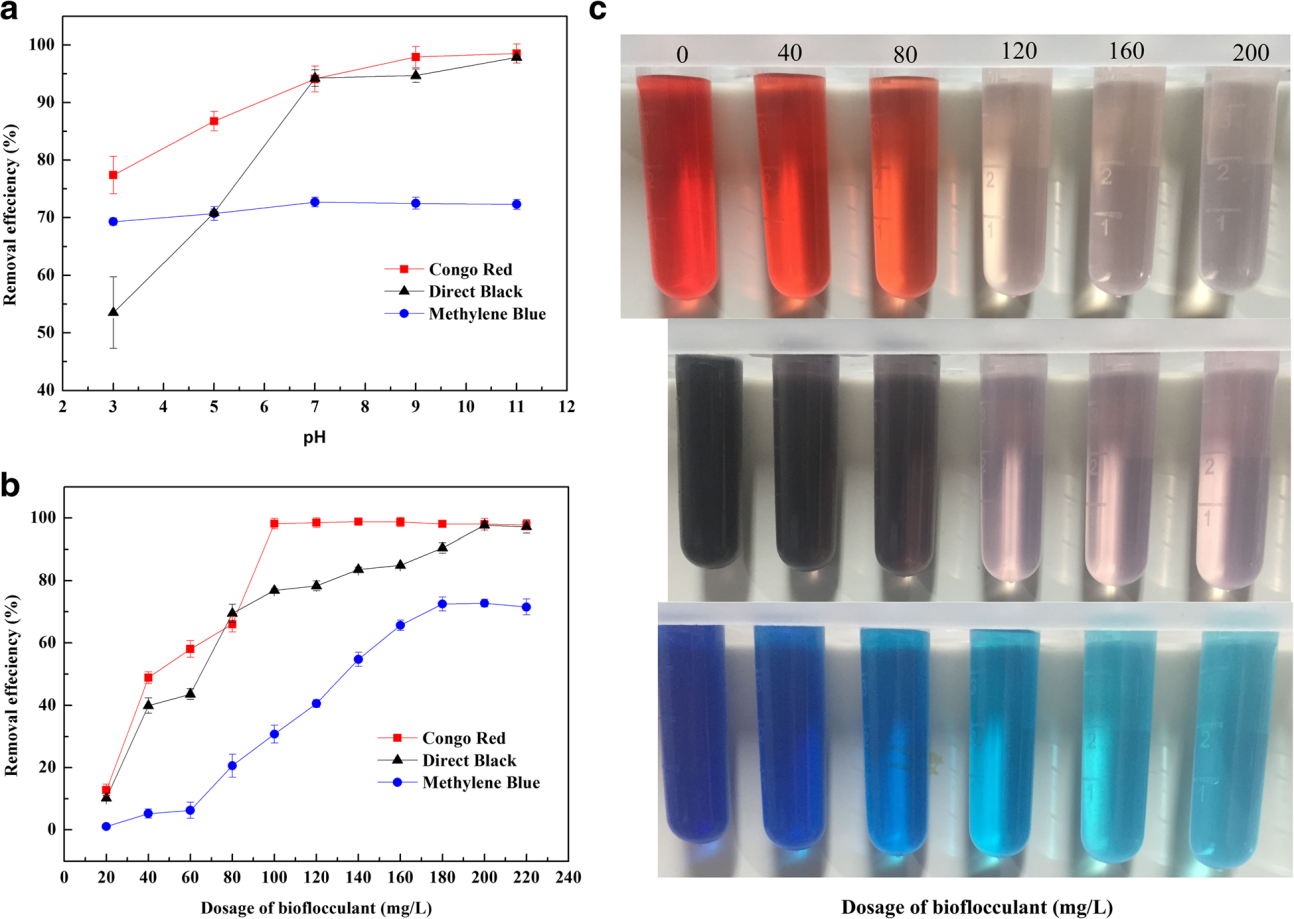
Effects of solution pH (**a**) and bioflocculant dosage (**b**) on dye removal efficiency

In the flocculation experiments, two anionic dyes (Congo Red and Direct Black) and one cationic dye (Methylene Blue) were used with different pH values. The results showed that the bioflocculant exhibited different decolourization capacity depending on the dye used and the solution pH (Fig. 6a). For anionic dye, a gradual increase in decoloration efficiency was observed from pH 3.0 to 11.0. The removal of anionic dyes was directly impacted by the availability and strength of positive charges in the solution, which in turn were fixed by the conformation and the cationicity of the bioflocculant. It can be explained by the theory reported by Somasundaran that pH influences the electrochemistry of the dyes and the dissociation of the polyelectrolytes, and hence their conformation in solution [50]. For cationic dye, the impact of pH on dye removal is not obvious, probably because of the cationic property of the bioflocculant. Overall, the bioflocculant had moderate removal ability for anionic dye, with the highest decolourization rates for Congo Red and Direct Black being 98.5% and 97.9%, respectively; a lower rate was observed when used with cationic dyes for Methylene Blue, at 72.7%. These results suggested that the bioflocculant was more effective for anionic dyes than cationic dyes. Similarly, the bioflocculant produced by *Kocuria rosea* was effective for the removal of anionic dyes [51]. In addition, the effect of mixing time on dye removal was investigated. The results showed that the dye removal efficiency after mixing the bioflocculant and dye for 5 min was identical to that after mixing for 10 min, which indicated that the adsorption of dye by the bioflocculant is a very rapid process.

#### Effect of Bioflocculant dosage on dye removal

The dye removal efficiency of bioflocculant at different adsorbent doses (20–220 mg/L) is shown in Fig. 6b, and a comparison of dye wastewater before and after flocculation is presented in Fig. 6c. Generally, the removal efficiency increased with the bioflocculant dosage, which is due to the increase in absorbent surface area and adsorbing sites. When the bioflocculant was administered, microflocs aggregated into larger ones due to the adsorption/bridging ability of the polysaccharides. The optimal bioflocculant dosages for Congo Red, Direct Black and Methylene Blue were 100, 200 and 180 mg/L, respectively. Further increasing the adsorbent resulted in a slight decrease in decolourization efficiency, since higher bioflocculant doses would inhibit small flocs from growing due to the stronger repulsion force between them [52]. Moreover, especially for anionic dyes, the bioflocculant exhibited excellent decolourization ability without the addition of any cationic salt.

## Conclusion

A bioflocculant-producing strain was isolated from seawater and identified as *Alteromonas* sp. CGMCC 10612. A maximum bioflocculant production of 11.18 g/L with a flocculating activity of 2575.4 U/mL was achieved in a 2-L fermenter. Its composition was predominantly polysaccharide (69.6%), which explains its thermal stability. Further, its high content of uronic acid (14.5%) indicated the presence of many functional groups containing nitrogen and oxygen atoms, which are preferred for flocculation. In addition, its high molecular weight (3.94 × 10^5^ Da) strengthens its competitive advantage in bridging function and flocculation ability. Above all, its excellent decolourization ability suggests its potential industrial utility for biotechnological processes.

## Additional files

Additional file 1: The phylogenetic tree of bioflocculant-producing strain H-6. (TIFF 52 kb)

Additional file 2: Effects of temperature (a) and initial pH (b) on bioflocculant production. (TIFF 1522 kb)

## Abbreviations

DO: Dissolved oxygen
EPS: Exopolysaccharide
FA: Flocculating activity
FTIR: Fourier Transform Infra-Red
HPGPC: High-performance gel permeation chromatography
NCBI: National center for biotechnological information
OD: Optical density
SEM: Scanning electron microscopy
