# Supplementary figures and images for: Characterization of a novel bioflocculant from a marine bacterium and its application in dye wastewater treatment
Source: BMC Biotechnol. 2017 Nov 17;17:84. doi: 10.1186/s12896-017-0404-z (PMC5693566; doi:10.1186/s12896-017-0404-z)

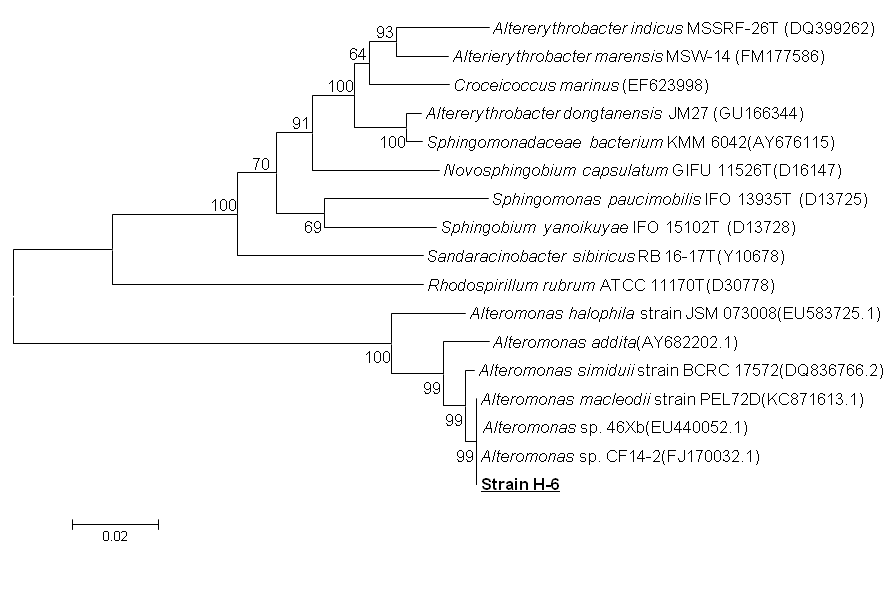

Supplement: Supplementary file 1 — The phylogenetic tree of bioflocculant-producing strain H-6. (TIFF 52 kb) [file 12896_2017_404_MOESM1_ESM.tif]

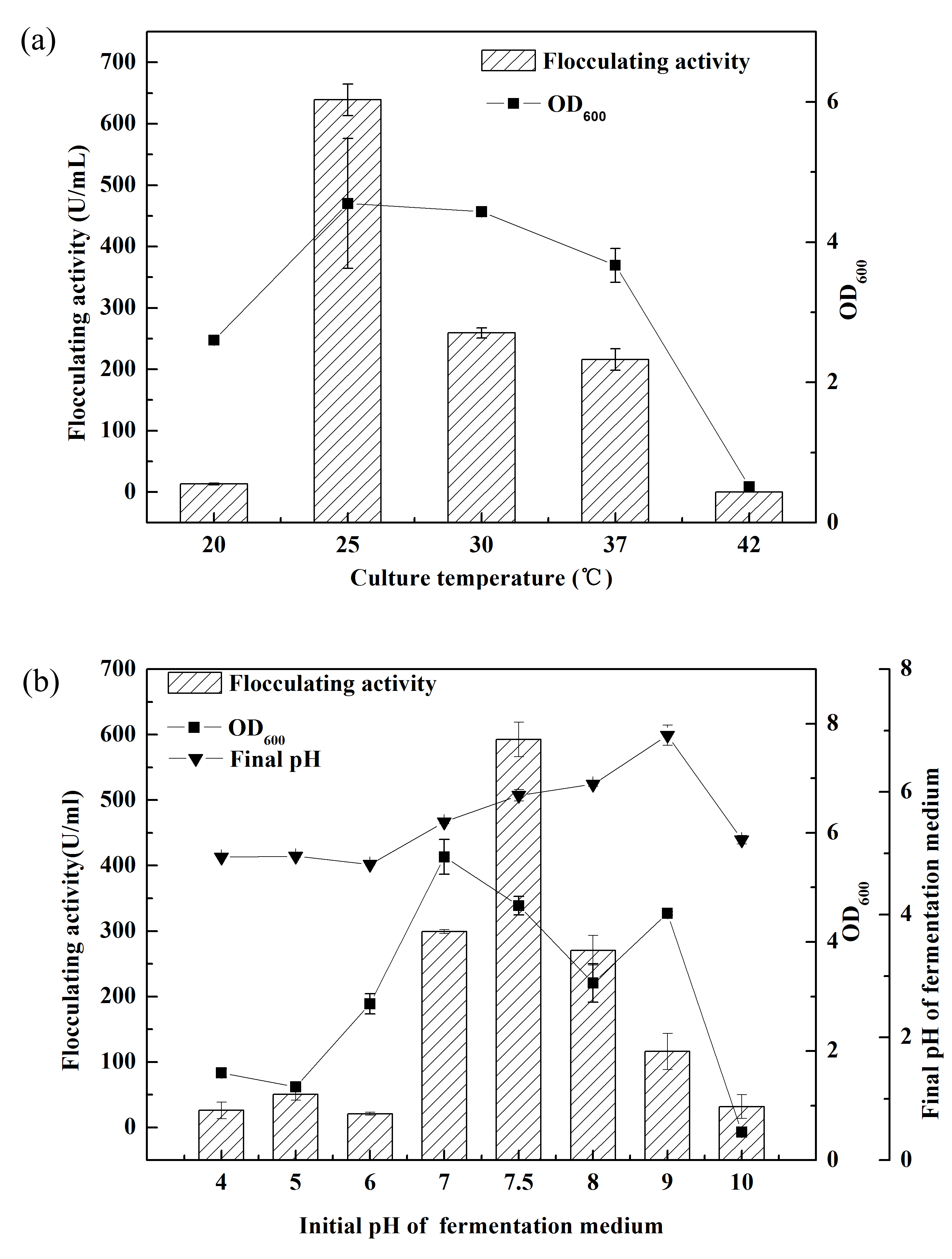

Supplement: Supplementary file 2 — Effects of temperature (a) and initial pH (b) on bioflocculant production. (TIFF 1522 kb) [file 12896_2017_404_MOESM2_ESM.tif]
